# Chronic pancreatitis and cancer risk in a matched cohort study using national claims data in South Korea

**DOI:** 10.1038/s41598-022-09426-z

**Published:** 2022-04-01

**Authors:** Minji Han, Thi Phuong Thao Tran, Jin-Kyoung Oh

**Affiliations:** 1 Division of Cancer Prevention, National Cancer Center, Goyang, Republic of Korea; 2 Department of Cancer Control and Population Health, National Cancer Center, Graduate School of Cancer Science and Policy, 323 Ilsan-ro, Ilsandong-gu, Goyang-si, Gyeonggi-do 410-769 Republic of Korea

**Keywords:** Epidemiology, Cancer epidemiology, Gastroenterology, Gastrointestinal diseases

## Abstract

This study aimed to examine the association between chronic pancreatitis (CP) and cancer incidence and mortality among the Korean population. Based on a cancer-free cohort of 8,317,616 individuals between 2002 and 2010, a matched cohort study was conducted, including 10,899 patients with CP, matched for sex and age with 32,697 individuals without CP. The case and control groups were followed up until the date of onset of cancer or death or the end of follow-up date (December 31, 2018). Cox proportional hazards regression was performed to assess the risk of cancer incidence and mortality. Compared to the control group, patients with CP had a higher risk of all cancers with a hazard ratio (HR) of 1.2 [95% confidence interval (CI) 1.1–1.3]. CP was associated with an increased risk of esophageal cancer (HR 3.9, 95% CI 1.8–8.5) and pancreatic cancer (HR 3.9, 95% CI 2.7–5.5) and a decreased risk of colorectal cancer (HR 0.7, 95% CI 0.5–0.9). Regarding cancer mortality, patients with CP had a 1.2-fold risk of all cancer mortality (95% CI 1.1–1.4), compared with the control group. Patients with CP had a higher risk of death from esophageal cancer (HR 3.5, 95% CI 1.5–8.0) and pancreatic cancer (HR 3.3, 95% CI 2.3–4.7) but had a lower risk of death due to stomach cancer (HR 0.4, 95% CI 0.2–0.8). Patients with CP had a higher risk for both incidence and mortality of all cancer types, especially pancreatic and esophageal cancers, compared with the sex- and age-matched control group.

## Introduction

Chronic pancreatitis (CP) is a syndrome characterized by chronic progressive fibro-inflammation and scarring with parenchymal calcifications with a symptom of severe abdominal pain and eventually leads to irreversible damage of exocrine and endocrine pancreatic ducts, cancer, and other complications^1^. The global incidence of CP was 9.62 cases [95% confidence interval (CI) 7.86–11.78] per 100,000 person-years (PYs) and was twice higher in men compared to women^2^. A systematic analysis in 195 countries and territories revealed that the age-standardized prevalence and years lived with disability of overall pancreatitis increased between 1990 and 2017^3^. Therein, the evidence on an increasing trend of CP prevalence was also observed in several countries^4,5^. Thus, CP has become a critical public health problem.

CP is a well-known risk for pancreatic cancer, as observed in previous studies^6–14^. Findings from a meta-analysis of seven cohort and case–control studies suggested that patients with chronic pancreatitis were more likely to have a 13.3-fold higher risk of pancreatic cancer development (95% CI 6.1–28.9)^15^. Another meta-analysis also confirmed that patients with CP were strongly associated with an increased risk of pancreatic cancer [pooled odds ratio (OR) 10.35, 95% CI 9.13–11.75]^6^. A systematic review and meta-analysis by Kirkegård et al. also revealed that the pooled effect estimates for pancreatic cancer in patients with CP were 16.16 (95% CI 12.59–20.73)^7^. Despite unclear mechanisms of pathogenesis for the association between pancreatitis and pancreatic cancer^6^, several hypothesized molecular pathways have been formulated related to inflammation recurrence^16,17^ or genetic mutation^18,19^.

Furthermore, several studies have suggested an association between CP and the development of various types of cancer, including those of the bile duct^20^, colon^12,21^, gastric^21^, thyroid^11^, breast^12^, ovarian^12^ bladder cancers^12^ and non-Hodgkin lymphoma^12,22^. However, the evidence was produced among a limited number of patients with CP. Another larger matched cohort study comprising 11,237 patients with CP in Taiwan revealed novel evidence that patients with CP also had a higher likelihood of developing head and neck cancers (adjusted HR 1.31, 95% CI 1.07–1.60) compared with those without CP^23^. However, this study did not examine the effect of CP on the risk of other cancers.

Regarding mortality, a higher HR of 1.4 (95% CI 1.3–1.5) for cancer-related death was found for Danish patients with CP compared with those without CP^10^. Another evidence from South Korea also demonstrated that CP was significantly associated with 5-year cancer mortality^5^. However, the risk of cancer-specific mortality for CP has not yet been investigated in these two studies.

CP is a severe clinical condition that can cause severe complications, including cancer; however, the scientific evidence on their association is sparse because of a lack of epidemiological data on CP in the general population, especially in Asia^13^. Meanwhile, the highest number of prevalent and incident cases of pancreatitis was found in East Asia^3^, which could threaten the pancreatitis-related public health, including cancer burden, in these countries. In South Korea, a 13-year cohort study showed an increasing trend of CP, and the prevalence of CP increased from 90/100,000 individuals in 2002 to 560/100,000 individuals in 2015^5^. Moreover, considering that the association between excessive alcohol consumption and CP was demonstrated in Western countries^1^, the risk for CP would be elevated owing to increased drinking prevalence (ie, consumed at least once a month) in Korea, accounting for 74.0% and 50.5% in men and women, respectively, in 2017^24^. Therefore, this was the first study to examine the association between CP and cancer incidence and mortality in South Korea using data from a nationwide retrospective cohort study.

## Results

Our matched cohort study included 10,899 patients with CP, matched for sex and age with 32,697 individuals without CP. The mean age of the participants was 49.9 years (standard deviation = 12.7), and 72.9% of them were men. Regarding baseline characteristics, the CP group had a higher proportion of current smokers, consumed a higher amount of alcohol per day, had less frequency of physical activity, had higher percentage of normal and underweight people, and had higher blood sugar level and CCI score than the control group (Table 1).

**Table 1:** Baseline characteristics of the study population in 2002–2003 based on the case and control groups.

	Case group (n = 10,899)	Control group (n = 32,697)	*p*-value
	n (%)	n (%)	
Age, mean (SD)	49.92 (12.71)	49.92 (12.71)	1
**Sex**			1
Men	7947 (72.91)	23,841 (72.91)	
Women	2952 (27.08)	8856 (27.08)	
**Income level**			< 0.0001
High (Q4)	3611 (33.13)	12,524 (38.30)	
Middle high (Q3)	2911 (26.70)	8340 (25.50)	
Middle low (Q2)	2473 (22.69)	6430 (19.66)	
Low (Q1)	1904 (17.46)	5403 (16.52)	
**Smoking status (missing = 960)**			< 0.0001
Never	5285 (48.49)	17,459 (53.39)	
Ex-smoker	1087 (9.973)	3948 (12.07)	
Current smoker	4323 (39.66)	10,534 (32.21)	
**Alcohol consumption per day (gram/day, missing = 981)**			< 0.0001
0	4547 (41.71)	14,764 (45.15)	
< 12.5	3240 (29.72)	11,380 (34.80)	
12.5–50	2361 (21.66)	5145 (15.73)	
50	536 (4.92)	642 (1.96)	
**Frequency of physical activity (missing = 1549)**			< 0.0001
More than 5 per week	942 (8.642)	2782 (8.508)	
1~4 per week	3576 (32.81)	11,713 (35.82)	
None	6038 (55.39)	16,996 (51.98)	
**Body mass index (kg/m**^**2**^**, missing = 65)**			< 0.0001
Normal or underweight (< 23)	5189 (47.60)	12,469 (38.13)	
Overweight (23–24.9)	2629 (24.12)	8894 (27.20)	
Obesity (25 +)	3064 (28.11)	11,286 (34.51)	
**Fasting Blood Sugar (mg/dL, missing = 84)**			< 0.0001
Normal (< 100)	6973 (63.97)	22,927 (70.11)	
Prediabetes (100–125)	2607 (23.91)	7353 (22.48)	
Diabetes (126 +)	1296 (11.89)	2356 (7.21)	
**Total cholesterol (mg/dL, missing = 100)**			< 0.0001
Normal (< 200)	6283 (57.64)	17,876 (54.67)	
Borderline high (200–239)	3188 (29.25)	10,484 (32.06)	
High (240 +)	1393 (12.78)	4272 (13.06)	
CCI* (missing = 457)			< 0.0001
0	9974 (91.51)	31,992 (97.84)	
≥ 1	640 (5.87)	533 (1.63)	

*CCI, Charlson Comorbidity Index, among inpatients only at baseline in 2002–2003.

*SD* standard deviation.

At the end of follow-up, there were 736 (6.75%) and 1879 (5.75%) incident cancer cases in the case and control groups, respectively. Compared with the control group, the CP group had a higher risk of all cancers, with an HR of 1.2 (95% CI 1.1–1.3) after adjusting for other covariates. By specific cancer types, CP was associated with increased risk of esophageal cancer (HR 3.9, 95% CI 1.8–8.5) and pancreatic cancer (HR 3.9, 95% CI 2.7–5.5), whereas it was associated with decreased risk of colorectal cancer (HR 0.7, 95% CI 0.5–0.9) (Table 2). Similar results were observed for cancer-related mortality. The CP group had a higher (1.2-fold) risk of cancer mortality compared with the control group. With respect to specific cancer types, the CP group had a higher risk of death from esophageal cancer (HR 3.5, 95% CI 1.5–8.0) and pancreatic cancer (HR 3.3, 95% CI 2.3–4.7) compared with the control group. Additionally, the CP group had a lower risk of death due to stomach cancer (HR 0.4, 95% CI 0.2–0.8) than the control group (Table 3).

**Table 2:** Hazard ratios of cancer incidence.

Cancer types	ICD-10	Cancer cases		Crude HR		Adjusted HR^a^	
		Case group (n = 10,899)	Control group (n = 32,697)	HR (95% CI)	*p*-value	HR (95% CI)	*p*-value
		n (%)	n (%)				
All	C00–97	736 (6.75)	1879 (5.75)	1.3 (1.2–1.4)	< 0.001	1.2 (1.1–1.3)	< 0.001
Lip, oral cavity and pharynx	C00–14	17 (0.16)	28 (0.09)	2.0 (1.1–3.6)	0.029	1.7 (0.9–3.2)	0.103
Esophagus	C15	19 (0.17)	11 (0.03)	5.6 (2.7–11.7)	< 0.001	3.9 (1.8–8.5)	< 0.001
Stomach	C16	102 (0.94)	337 (1.03)	1.0 (0.8–1.2)	0.846	1.0 (0.8–1.2)	0.710
Colon and rectum	C18–20	61 (0.56)	275 (0.84)	0.7 (0.5–0.9)	0.019	0.7 (0.5–0.9)	0.006
Liver^b^	C22	68 (0.62)	140 (0.43)	1.6 (1.2–2.1)	0.002	1.1 (0.8–1.5)	0.438
Gallbladder and biliary tract	C23–24	34 (0.31)	67 (0.2)	1.6 (1.1–2.5)	0.019	1.5 (0.9–2.2)	0.094
Pancreas	C25	74 (0.68)	62 (0.19)	3.9 (2.8–5.4)	< 0.001	3.9 (2.7–5.5)	< 0.001
Larynx	C32	8 (0.07)	13 (0.04)	2.0 (0.8–4.8)	0.128	1.5 (0.6–3.8)	0.375
Lung	C33–34	102 (0.94)	219 (0.67)	1.5 (1.2–1.9)	0.001	1.2 (0.9–1.5)	0.170
Breast	C50	23 (0.21)	66 (0.2)	1.1 (0.7–1.8)	0.622	1.1 (0.7–1.8)	0.747
Cervix uteri	C53	6 (0.06)	9 (0.03)	2.2 (0.8–6.1)	0.507	2.3 (0.8–6.5)	0.115
Corpus uteri	C54	1 (0.01)	3 (0.01)	1.1 (0.1–10.5)	0.941	1.3 (0.1–15.2)	0.844
Ovary	C56	3 (0.03)	6 (0.02)	1.6 (0.4–6.4)	0.507	2.2 (0.5–9.4)	0.273
Prostate	C61	69 (0.63)	207 (0.63)	1.1 (0.8–1.4)	0.573	1.1 (0.8–1.4)	0.675
Testis	C62	0 (0.00)	1 (0.003)	0.0	0.997	0.0	0.999
Kidney	C64	13 (0.12)	27 (0.08)	1.6 (0.8–3.0)	0.184	1.5 (0.7–3.0)	0.262
Bladder	C67	22 (0.2)	60 (0.18)	1.2 (0.7–1.9)	0.490	1.2 (0.7–2.0)	0.464
Brain	C70–72	5 (0.05)	19 (0.06)	0.8 (0.3–2.3)	0.744	0.7 (0.2–2.1)	0.542
Thyroid gland	C73	34 (0.31)	139 (0.43)	0.8 (0.5–1.1)	0.206	0.9 (0.6–1.4)	0.712
Hodgkin lymphoma	C81	1 (0.01)	0 (0.00)	N.A	N.A	N.A	0.999
Non-Hodgkin lymphoma	C82–86,96	4 (0.04)	9 (0.03)	1.4 (0.4–4.6)	0.552	1.3 (0.6–2.6)	0.498
Multiple myeloma and malignant plasma cell neoplasm	C90	8 (0.07)	23 (0.07)	1.1 (0.5–2.5)	0.785	1.2 (0.4–4.0)	0.780
Leukemia	C91–95	14 (0.13)	29 (0.09)	1.6 (0.8–3.0)	0.166	1.0 (0.4–2.3)	0.971
Others	Others	48 (0.44)	129 (0.39)	1.2 (0.9–1.7)	0.268	1.0 (0.7–1.5)	0.808

^a^Adjusted hazard ratio was adjusted for smoking status, alcohol consumption, physical activity, body mass index, fasting blood sugar level, total cholesterol level, and Charlson Comorbidity Index score.

^b^Liver cancer was additionally adjusted for chronic infection of virus B and C hepatitis (International Classification of Disease 10th edition: B18).

*N.A.* not available.

**Table 3:** Hazard ratios of cancer mortality.

Cancer types	ICD-10	Cancer death		Crude HR		Adjusted HR^a^	
		Case group	Control group	HR (95% CI)	*p*-value	HR (95% CI)	*p*-value
		n (%)	n (%)				
All	C00–97	365 (3.35)	788 (2.41)	1.5 (1.3–1.7)	< 0.001	1.2 (1.1–1.4)	< 0.001
Lip, oral cavity and pharynx	C00–14	7 (0.06)	8 (0.02)	2.8 (1.0–7.8)	0.043	2.2 (0.7–6.2)	0.135
Esophagus	C15	14 (0.13)	12 (0.04)	3.8 (1.8–8.2)	< 0.001	3.5 (1.5–8.0)	< 0.001
Stomach	C16	17 (0.16)	102 (0.31)	0.5 (0.3–0.9)	0.018	0.4 (0.2–0.8)	0.007
Colon and rectum	C18–20	20 (0.18)	62 (0.19)	1.0 (0.6–1.7)	0.848	0.9 (0.5–1.6)	0.929
Liver^b^	C22	60 (0.55)	98 (0.30)	1.7 (1.2–2.4)	0.001	1.3 (0.9–1.9)	0.062
Gallbladder and biliary tract	C23–24	21 (0.19)	61 (0.19)	1.1 (0.7–1.8)	0.650	0.9 (0.5–1.6)	0.851
Pancreas	C25	71 (0.65)	68 (0.21)	3.4 (2.4–4.7)	< 0.001	3.3 (2.3–4.7)	< 0.001
Larynx	C32	1 (0.01)	2 (0.01)	1.6 (0.1–18.2)	0.684	0.9 (0.0–12.7)	0.997
Lung	C33–34	101 (0.93)	219 (0.67)	1.5 (1.2–1.9)	< 0.001	1.2 (0.9–1.5)	0.221
Breast	C50	1 (0.01)	4 (0.01)	0.8 (0.1–7.3)	0.857	0.9 (0.1–8.2)	0.938
Cervix uteri	C53	0 (0.00)	3 (0.01)	0.0	0.995	0.0	0.998
Corpus uteri	C54	0 (0.00)	0 (0.00)	N.A	N.A	N.A	N.A
Ovary	C56	1 (0.01)	6 (0.02)	0.5 (0.1–4.5)	0.570	0.0	0.997
Prostate	C61	4 (0.04)	20 (0.06)	0.7 (0.2–1.9)	0.438	0.7 (0.2–2.2)	0.569
Testis	C62	0 (0.00)	0 (0.00)	N.A	N.A	N.A	N.A
Kidney	C64	6 (0.06)	13 (0.04)	1.5 (0.6–4.0)	0.408	1.3 (0.4–3.7)	0.679
Bladder	C67	4 (0.04)	17 (0.05)	0.8 (0.3–2.3)	0.632	0.7 (0.2–2.1)	0.514
Brain	C70–72	2 (0.02)	8 (0.02)	0.8 (0.2–3.8)	0.792	0.8 (0.1–3.7)	0.770
Thyroid gland	C73	1 (0.01)	5 (0.02)	0.7 (0.0–5.6)	0.701	0.6 (0.0–5.5)	0.683
Hodgkin lymphoma	C81	1 (0.01)	1 (0.003)	3.1 (0.1–50.7)	0.415	2.8 (0.2–47.1)	0.476
Non-Hodgkin lymphoma	C82–86,96	7 (0.06)	15 (0.05)	1.5 (0.6–3.6)	0.369	1.2 (0.5–3.2)	0.720
Multiple myeloma and malignant plasma cell neoplasm	C90	4 (0.04)	8 (0.02)	1.6 (0.4–5.3)	0.431	1.5 (0.4–5.2)	0.499
Leukemia	C91–95	3 (0.03)	17 (0.05)	0.5 (0.1–1.9)	0.372	0.5 (0.1–1.7)	0.247
Others	Others	19 (0.17)	39 (0.12)	1.6 (0.9–2.7)	0.103	1.5 (0.8–2.6)	0.169

^a^Adjusted hazard ratio was adjusted for smoking status, alcohol consumption, physical activity, body mass index, fasting blood sugar level, total cholesterol level, and Charlson Comorbidity Index.

^b^Liver cancer was additionally adjusted for chronic infection of virus B and C hepatitis (International Classification of Disease 10th edition: B18).

*N.A.* not available.

**Figure 1:**
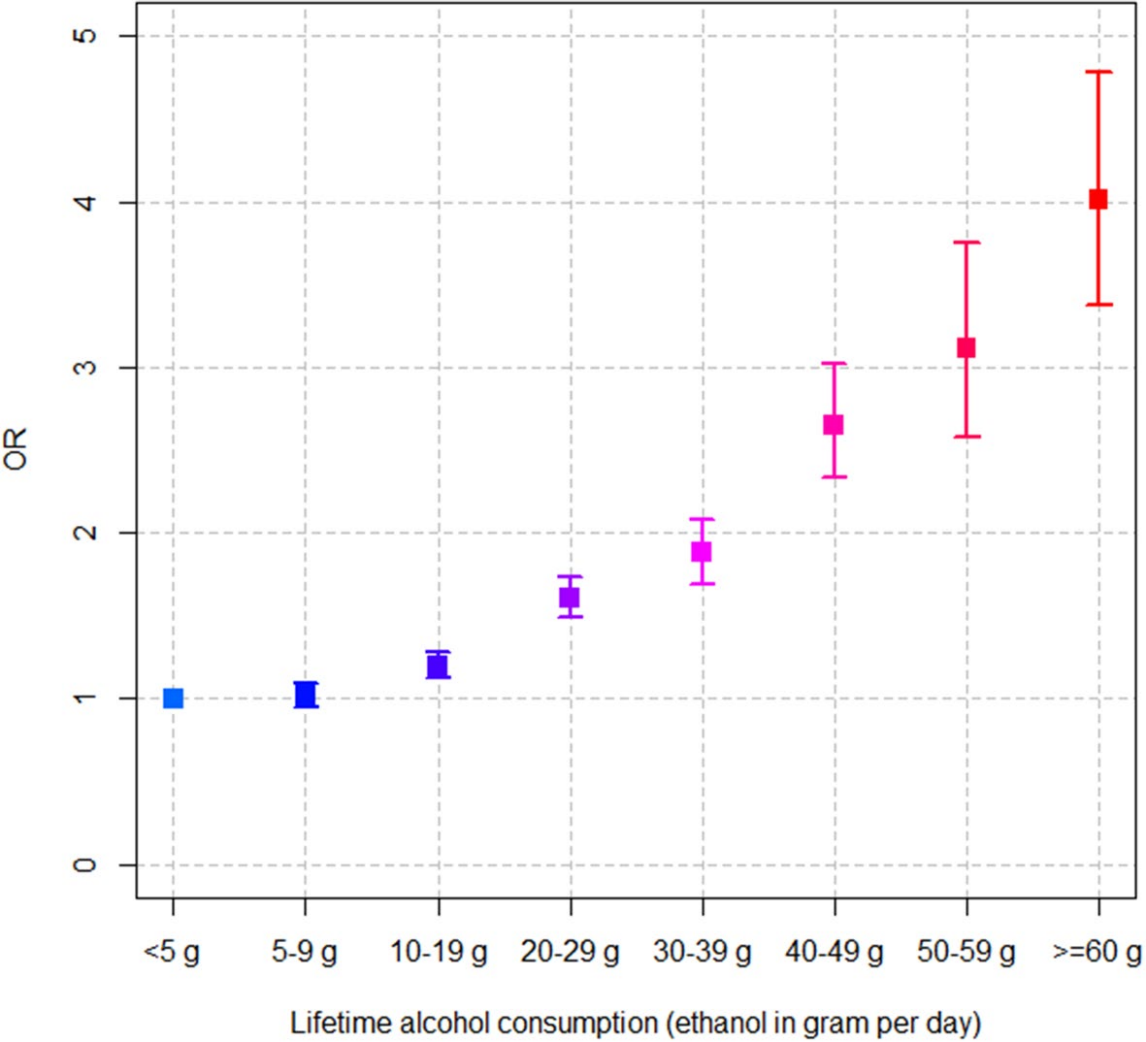
Association between lifetime alcohol consumption and chronic pancreatitis.

Furthermore, we examined the association between CP and alcohol consumption (Supplemental Table 1). This finding suggested that there was a clear dose–response association between alcohol consumption and CP. In particular, the higher the amount of alcohol consumption, the higher the risk of CP. Individuals who consumed ≥ 60 g ethanol daily had a four-time higher risk of CP compared with those who consumed the lowest alcohol level (Fig. 1).

## Discussion

This is the first matched cohort study in Asia that included a remarkably high number of patients with CP using a nationwide population database to examine the association between CP and various specific cancer incidences and mortality. The study revealed evidence that Korean patients with CP had a 1.2 times higher risk of both overall cancer incidence and mortality compared with the sex- and age-matched control group. To date, there is only one large-scale study in Denmark, comprising 11,972 patients with CP and 119,720 controls, that also investigated the risk of cancer incidence and mortality in patients with CP. The results suggested that the HR of all cancers was 1.2 (95% CI 1.1–1.3), similar to findings from our study. Risk of cancer-related death was slightly higher than that in our study, associated with an HR of 1.4 (95% CI 1.3–1.5)^10^. Considering the insufficient epidemiological studies in this field worldwide, especially in Asia, our finding could serve as a novel scientific reference to fill the knowledge gap for effective screening, surveillance, and prevention of cancer in patients with CP.

CP is strongly associated with an increased risk of pancreatic cancer incidence and mortality. In 1993, the first large cohort study comprising 1552 patients with CP from six countries initially demonstrated evidence on the significant association between pancreatitis and the development of pancreatic cancer^25^. In that study, CP was significantly related to pancreatic cancer, yielding a standardized incidence ratio (SIR) of 26.3 (95% CI 19.9–34.2). In 1997, Karlson et al. also observed a high risk of pancreatic cancer among 4546 patients with CP with SIR of 7.6 (95% CI 6.0–9.7). However, the authors also clarified that behavioral risk factors, including smoking or alcohol consumption, could be a bias contributing to pancreatic cancer^26^, which was also mentioned in a later study by Goldacre in 2008^27^. Since these earlier studies had a significantly small number of cancer cases, large cohort studies have been required to clarify the association between pancreatic cancer and CP. Thereafter, a nationwide matched cohort study by Bang et al., who obtained data from the Danish Registry from 1995 to 2010, revealed the strong association between CP and pancreatic cancer with an HR of 6.9 (95% CI 5.6–8.6), but this finding did not adjust for risk behaviors, such as smoking and drinking^10^. Considering all the limitations of prior studies, in the present study, we considered all potential confounders, ie, not only sociodemographic factors and comorbidity but also lifestyle behaviors, including smoking, alcohol consumption, physical activity, and BMI. The results confirmed that CP was strongly associated with pancreatic cancer incidence and mortality, with HRs of 3.9 (95% CI 2.7–5.5) and 3.3 (95% CI 2.3–4.7), respectively. Although their pathomechanism is not fully understood^6^, it has been discussed widely in the literature^9^. The association between CP and pancreatic cancer is probably due to the recurrence of inflammation that could stimulate pancreatic stellate cells, possibly leading to carcinogenesis^16,17^. Additionally, KRAS oncogene was commonly found in patients with CP and is present in almost all pancreatic cancers (90%), harboring mutations^17^. Moreover, KRAS mutation could play a crucial role in pancreatic cancer development in patients with CP in a meta-analysis of 15 studies^18^. Recently, SPINK1 mutations have been proposed to be related to nonalcoholic CP that is probably associated with the earlier onset of pancreatic cancer^9^. In clinical practice, the onset of pancreatic cancer is unknown in patients with CP. Furthermore, pancreatic cancer is commonly diagnosed late, and its symptoms and clinical indices of both conditions usually resemble CP, which poses a challenge for effective surgery^8^. In particular, more than half of pancreatic cancers are diagnosed at an advanced stage. In men especially, only 9.9% are diagnosed at the localized stage, whereas 48.8% are diagnosed at an advanced stage^28^, resulting in low survival rates. The recent 5-year relative survival rate of pancreatic cancer is 12.6%, whereas it has been significantly improved up to 70.3% for all cancers in South Korea^28^. In fact, pancreatic cancer screening is not recommended for average-risk individuals because it could cause more harm than good^19^; however, high-risk individuals who have CP predisposed to hereditary pancreatitis should be screened for improved outcomes from pancreatic cancer surveillance^29^.

In Western countries, excessive alcohol consumption was the most important risk factor for CP^1,30^, which contributed to approximately 65% of all CP cases^31^. The dose–response association between CP and alcohol consumption was clearly determined in a systematic review and meta-analysis^32^, which was also observed in our study. In consideration of the change in behavior over time, we assessed the dose–response association between CP and alcohol consumption, which was measured the lifetime average consumption collected from multiple health examinations. In fact, South Korea is a country where alcoholic beverages are widely consumed, exacerbating the risk of alcohol-related pancreatitis^24^. The mechanism of pathogenesis of alcohol-induced damage of the pancreas is unclear, but one proposed hypothesis is that oxidative stress induced by alcohol metabolites directly damages the gland^31^. Moreover, the interaction of various pathways of alcohol metabolism with other risk factors, such as genetic, dietary, environmental, and lifestyle-related factors, could be initially activated, which could eventually be associated with cancer development through the multistage process of carcinogenesis^9^. Hence, it could be postulated that both CP and esophageal cancer outcomes share risk factors, such as alcohol consumption and smoking. Additionally, chronic progressive inflammation in CP could enhance the process of inflammation-associated carcinogenesis^23^, which could explain the significant association between CP and esophageal cancer in our study. In particular, the proportion of patients with CP who smoked and consumed alcohol was significantly higher than that in the control group, which could contribute to the increased risk of esophageal disease among patients with CP.

Furthermore, the significant association between CP and other cancers, such as liver and lung cancers, was suggested by a large study by Bang et al. in Denmark^10^, which was consistent with our results in crude HR. However, the significant effect did not remain after adjusting for lifestyle behavior confounders in our study. Therefore, insufficient information on behavior-related behaviors in the study by Bang et al. could be a reason for the inconsistency between the findings of the two studies. In contrast, CP seemingly lessened the risk of colon cancer, which was suggested in our study as well as in that of Bang et al^10^. It could be explained by the higher mortality rate in patients with CP, and its onset age was commonly sooner than 8 years compared with colorectal cancer, thereby reducing the development of colorectal cancer^10^. A similar explanation could be proposed that CP was significantly associated with a decreased risk of death from stomach cancer, the most common cancer in Korea, as observed in our study.

Our study has some limitations. First, this was a retrospective cohort study using data from medical examinations. The characteristics of individuals who underwent medical checkups may differ from those who did not participate in the health examination. The participation rate in the general health examination among the eligible population was approximately 30% in 2002–2003, which covers a large Korean population^33^. Second, this study has a limitation stemming from its small number of CP cases. To ensure the causal association between CP and cancer, data of patients with CP were collected between 2002 and 2010; thus, the number of patients with CP decreased. Hence, the number of cancer cases was limited, especially in rare cancer types; therefore, statistical significance could not be observed in several specific cancer types in the survival analysis. Similarly, although alcohol consumption is the most important factor that contributes to the association between CP and cancer, stratified analysis by alcohol consumption status was not performed because of the insufficient number of cancer cases.

In conclusion, this nationwide matched cohort study in South Korea suggested that patients with CP had a higher risk for both incidence and mortality of all cancer types, especially pancreatic and esophageal cancers, compared with the sex- and age-matched control group. We also observed a strong dose–response association between CP and alcohol consumption.

## Methods

### Data sources

We conducted a matched cohort study based on a nationwide population-based cohort using the database of the National Health Insurance Service (NHIS) in South Korea. The NHIS is a mandatory single-payer insurance that provides benefits for medical services. It is a universal coverage health insurance system for all citizens (approximately 50 million individuals). The NHIS provides a general health screening program for all insured adults biennially, free of charge. The participation rate in the general health screening program among the eligible population was 74.1% in 2019^34^. The cohort contains the sociodemographic, general health examination results, lifestyles and behaviors, and medical treatment, and their collection process is described elsewhere^35^. Data on 8,968,212 individuals who underwent health screening in 2002–2003 provided by the NHIS were used. A cancer-free cohort in 2010 comprising 8,317,616 individuals was created after excluding individuals who had missing information on sex and age (n = 42,638), aged 19 years or younger (n = 28,838), who had an error in death date (n = 573), who were diagnosed with cancer between 2002 and 2010 (n = 575,585), and who died from cancer between 2002 and 2010 (n = 2962).

### Matched cohort

**Figure 2:**
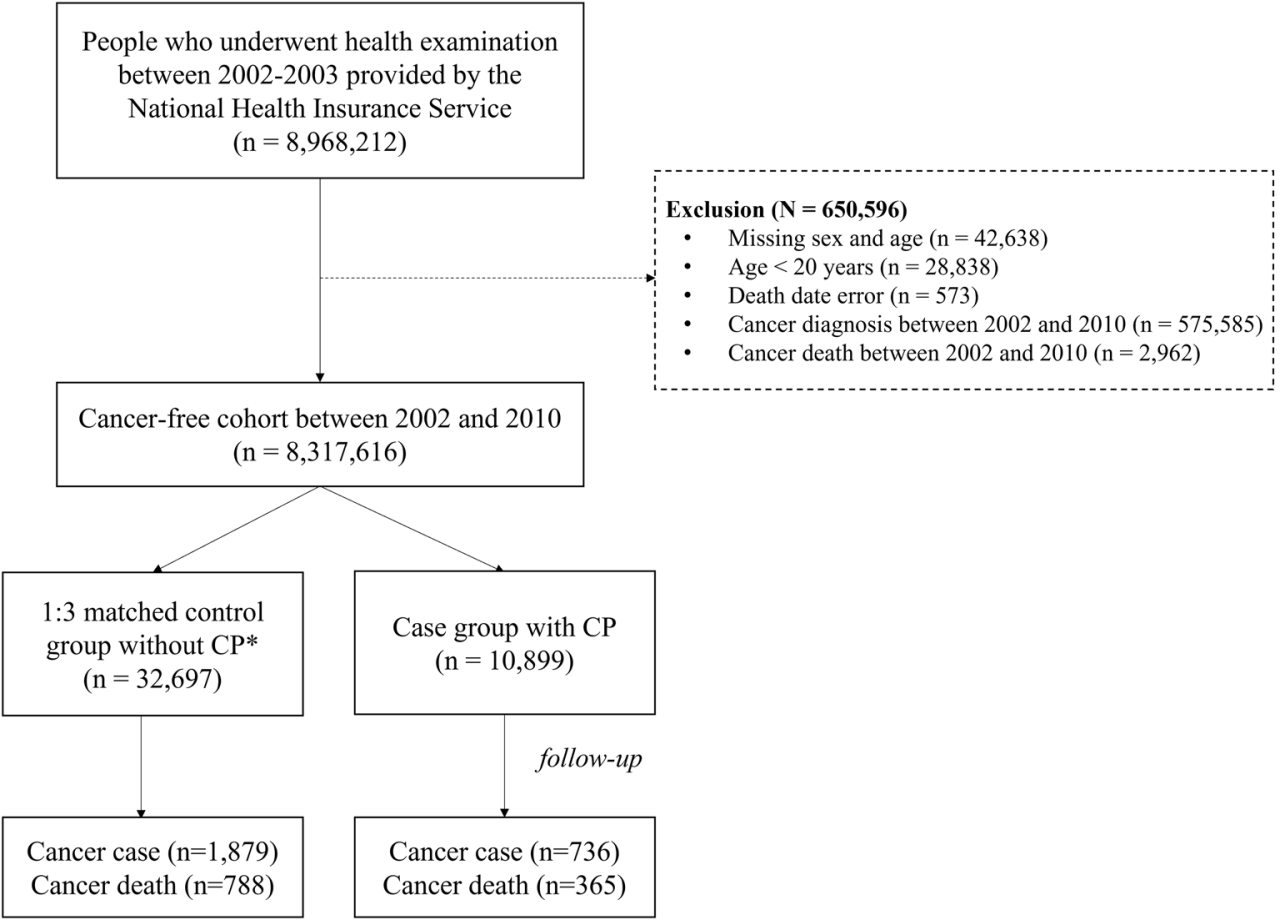
Flowchart of the study population. *Free of chronic pancreatitis for the whole study period.

In the cancer-free cohort, 10,899 individuals were newly diagnosed with CP between 2002 and 2010, using the International Classification of Disease 10th edition (ICD-10) codes K86.0 (alcohol-induced CP) and K86.1 (other CPs) as the primary diagnosis. We matched the case and control groups using propensity score matching considering age and sex. After calculating the propensity score through logistic regression analysis, three times the case (n = 32,697) was designated as the control among those without pancreatitis in the cancer-free cohort. A flowchart of the study population is shown in Fig. 2. As this study used anonymous secondary data, the study was exempted from review by the Institutional Review Board of the National Cancer Center, Korea (NCC2018-0279). This study was conducted according to the Declaration of Helsinki.

### Outcomes and follow-up

The ICD-10 codes corresponding to cancer (C00-C97) as a primary diagnosis combined with a special code for cancer claims (V193, V194, and V027) were used to identify cancer occurrence during the follow-up period. In the claims data, these special codes indicate a confirmed cancer diagnosis for expanded benefit coverage. Cancer death was also ascertained using cause of death provided by the Statistics Korea^36^. Based on the ICD-10 codes, we evaluated the incidence and mortality of all cancer types (C00-C97) and other specific cancers, including lip cancer, oral cavity and pharyngeal cancer (C00-C14), esophageal cancer (C15), stomach cancer (C16), colon and rectal cancer (C18–20), liver cancer(C22), gallbladder and biliary tract cancer (C23, C24), pancreatic cancer (C25), laryngeal cancer (C32), lung cancer (C33, C34), breast cancer (C50), cervix uteri cancer (C53), corpus uteri cancer (C54), ovarian cancer (C56), prostate cancer (C61), testicular cancer (C62), kidney cancer (C64), bladder cancer (C67), brain cancer (C70–C72), thyroid gland cancer (C73), Hodgkin lymphoma (C81), multiple myeloma and malignant plasma cell neoplasm (C90), leukemia (C91–C95), and non-Hodgkin lymphoma (C82–C86, C96). The CP and control groups were followed up until the date of onset of cancer, death, or end of follow-up date (December 31, 2018).

### Covariates

Covariates included sociodemographic (ie, age, sex, and income level), lifestyle and risk behaviors, and disease-related variables, which were retrieved from the baseline 2002–2003. Lifestyle and risk behaviors included smoking status (never smoker, former smoker, or current smoker), frequency of physical activity (none, 1–4 times, or ≥ 5 times per week), and body mass index (BMI) (< 23 kg/m^2^, normal and underweight; 23–24.9 kg/m^2^, overweight, or ≥ 25 kg/m^2^, obesity) according to the World Health Organization obesity standard for the Asian population^37^. Alcohol consumption per day was calculated based on the frequency of alcohol consumption multiplied by the number of alcohol drinks per day (amount of alcohol from soju using standard ethanol concentration 20% in one bottle of soju). It was categorized into four groups as 0, < 12.5, 12.5–49, or ≥ 50 ethanol in grams per day^38^. Lifetime alcohol consumption was calculated by the mean of alcohol grams per day collected from multiple health examinations during the study period between 2002 and 2018. It was classified into < 5, 5–9, 10–19, 20–29, 30–39, 40–49, 50–59, and ≥ 60 g. Serum biochemical parameters, including fasting blood sugar (< 100 mg/dL, normal; 100–125 mg/dL, prediabetes; or ≥ 126 mg/dL, diabetes) and total cholesterol (< 200 mg/dL, normal; 200–239 mg/dL, borderline high; ≥ 240 mg/dL, high), were measured. The Charlson Comorbidity Index (CCI) was also calculated for inpatients using ICD-10 codes^39^, categorized into 0 or ≥ 1 score.

### Statistical analyses

Characteristics of the study population were displayed as frequencies with percentages for categorical variables and as means with standard deviations for numerical variables. The chi-squared test was used to assess statistical differences in baseline characteristics between the case and control groups. PYs were defined as the time from cohort entry until the presence of cancer, cancer death, death, or end of follow-up date (December 31, 2018). We calculated the cancer incidence as the number of cancers divided by the total number of PYs. Cox proportional hazards regression was performed to assess the risk of total cancer incidence and mortality. In the multivariate model, we adjusted for age, sex, income level, smoking status, alcohol consumption per day, frequency of physical activity, BMI, fasting blood sugar level, total cholesterol level, and CCI score. Additionally, the hazard ratio (HR) for liver cancer was adjusted by infection of virus B and C hepatitis (ICD-10: B18) in the multivariate model. The number of observations in the multivariate model was reduced owing to missing information on covariates. Furthermore, logistic regression was performed to assess the association between CP and lifetime drinking; thus, the ORs and CIs were calculated. All statistical analyses were performed using SAS version 9.4 (SAS Institute, Inc., Cary, NC), and figures were visualized using R Studio 4.0.3 (RStudio, PBC, Boston, MA).

## Supplementary Information

Supplementary Information.

## Data Availability

This study used data from the National Health Information Database (NHIS-2020-1-237), provided by the NHIS. The datasets generated and analyzed during the current study are available upon request from the National Health Insurance Sharing Service, https://nhiss.nhis.or.kr/.

## Abbreviations

CCI: Charlson comorbidity index
CI: Confidence interval
CP: Chronic pancreatitis
HR: Hazard ratio
ICD-10: International classification of disease 10th edition
NHIS: National Health Insurance Service
PYs: Person-years
