# Supplementary material for: Chronic pancreatitis and cancer risk in a matched cohort study using national claims data in South Korea
Source: Sci Rep. 2022 Apr 1;12:5545. doi: 10.1038/s41598-022-09426-z (PMC8975838; doi:10.1038/s41598-022-09426-z)
Supplement: Supplementary file 1 — Supplementary Information. [file 41598_2022_9426_MOESM1_ESM.docx]

Supplemental Table 1: Association between lifetime alcohol consumption and chronic pancreatitis

| Lifetime alcohol consumption | OR | 95%CI |
| --- | --- | --- |
| <5 g | 1 |  |
| 5-9 g | 1.02 | 0.95 - 1.09 |
| 10-19 g | 1.20 | 1.13 - 1.28 |
| 20-29 g | 1.61 | 1.49 - 1.73 |
| 30-39 g | 1.88 | 1.69 - 2.08 |
| 40-49 g | 2.65 | 2.33 - 3.02 |
| 50-59 g | 3.11 | 2.58 - 3.75 |
| ≥ 60 g | 4.01 | 3.37 - 4.78 |
